# Incomplete Inhibition of Sphingosine 1-Phosphate Lyase Modulates Immune System Function yet Prevents Early Lethality and Non-Lymphoid Lesions

**DOI:** 10.1371/journal.pone.0004112

**Published:** 2009-01-01

**Authors:** Peter Vogel, Michael S. Donoviel, Robert Read, Gwenn M. Hansen, Jill Hazlewood, Stephen J. Anderson, Weimei Sun, Jonathan Swaffield, Tamas Oravecz

**Affiliations:** Lexicon Pharmaceuticals, Inc. The Woodlands, Texas, United States of America; Oklahoma Medical Research Foundation, United States of America

## Abstract

**Background:**

S1PL is an aldehyde-lyase that irreversibly cleaves sphingosine 1-phosphate (S1P) in the terminal step of sphingolipid catabolism. Because S1P modulates a wide range of physiological processes, its concentration must be tightly regulated within both intracellular and extracellular environments.

**Methodology:**

In order to better understand the function of S1PL in this regulatory pathway, we assessed the *in vivo* effects of different levels of S1PL activity using knockout (KO) and humanized mouse models.

**Principal Findings:**

Our analysis showed that all S1PL-deficient genetic models in this study displayed lymphopenia, with sequestration of mature T cells in the thymus and lymph nodes. In addition to the lymphoid phenotypes, S1PL KO mice (S1PL^−/−^) also developed myeloid cell hyperplasia and significant lesions in the lung, heart, urinary tract, and bone, and had a markedly reduced life span. The humanized knock-in mice harboring one allele (S1PL^H/−^) or two alleles (S1PL^H/H^) of human S1PL expressed less than 10 and 20% of normal S1PL activity, respectively. This partial restoration of S1PL activity was sufficient to fully protect both humanized mouse lines from the lethal non-lymphoid lesions that developed in S1PL^−/−^ mice, but failed to restore normal T-cell development and trafficking. Detailed analysis of T-cell compartments indicated that complete absence of S1PL affected both maturation/development and egress of mature T cells from the thymus, whereas low level S1PL activity affected T-cell egress more than differentiation.

**Significance:**

These findings demonstrate that lymphocyte trafficking is particularly sensitive to variations in S1PL activity and suggest that there is a window in which partial inhibition of S1PL could produce therapeutic levels of immunosuppression without causing clinically significant S1P-related lesions in non-lymphoid target organs.

## Introduction

Sphingosine 1-phosphate (S1P), a breakdown product of sphingolipid metabolism [1]–[5], is present in all mammalian cells and serves as second messenger in signal transduction pathways that regulate cell differentiation, proliferation, and apoptosis -[1]–[13]. S1P is also released into the extracellular milieu by a variety of cell types, making it one of the most abundant biologically active lysophospholipids in circulation [2], [4], [14]. Autocrine and paracrine interactions between S1P and a family of G protein-coupled receptors (S1P1–S1P5) have been implicated in a wide range of physiological activities, including cardiovascular development and disease, neuronal cell survival, and immunity [1], [2], [4], [9], [10], [12], [14]–[16].

Systemic and local concentrations of S1P are regulated directly by three classes of enzymes within the sphingolipid pathway. Reversible synthesis of S1P is mediated by sphingosine kinases [2], [3], [10], which phosphorylate sphingosine to produce S1P, and by S1P phosphatases [4], [14], which remove the phosphate. Irreversible degradation of S1P is carried out by a single enzyme, Sphingosine 1-phosphate lyase (S1PL), which cleaves S1P into ethanolamine phosphate and a long chain aldehyde [4], [12]–[14], [17], [18]. S1PL is present in most mammalian cells except for platelets and erythrocytes [4], [14], [19], but its relative abundance varies by tissue and cell type.

The diverse effects of S1P make it difficult to dissect its role in different aspects of mammalian physiology and to predict the *in vivo* consequences of deficiency in S1P degrading enzymes, including S1PL. Disrupted lipid metabolism due to mutations in the catabolic enzymes of the sphingolipid pathway can result in sphingolipid storage diseases, which are characterized by the cellular storage and accumulation of the sphingolipid substrates [20]. The specific pathologic phenotype associated with each sphingolipid storage disease depends on both the expression pattern of the affected catabolic enzyme and the metabolic burden of each particular cell type. Although no human disease has been associated with S1PL deficiency, loss of S1PL in mice leads to stunted growth and early mortality, with defects reported in the kidney, bone, vasculature and myeloid cell lineages [21].

In the immune system, changes in local S1P concentration and gradient can alter inflammatory cell responses [22], [23], affect the barrier function of endothelial cells [24]–[26], and modify lymphocyte migration patterns [15], [16], [27]–[29]. Hematopoietic cell-specific knockdown of S1PL in the bone-marrow using RNA interference increased S1P levels in lymphoid organs and lead to lymphopenia after transplantation of the bone marrow into immunodeficient mice [28]. The described effect of S1PL knockdown is similar to that observed after treatment with the S1P-analog FTY720[29]. S1P and FTY720 elicit their effects on the immune system through interaction with the S1P1 receptor, which inhibits lymphocyte egress from primary and secondary lymphoid organs and in turn reduces the number of recirculating lymphocytes in peripheral blood [27], [30]–[35]. One physiological outcome of this systemic redistribution of lymphocytes is potent immunosuppression, which offers new opportunities for developing immunoregulatory agents to treat autoimmune and inflammatory diseases [36]–[40]. The S1P1 receptor is currently a target of pharmaceutical development, and enzymes of the S1P metabolic pathway may provide additional intervention points for improved therapeutic applications.

In this study, we determined the levels of S1PL enzyme activity in gene knockout and humanized mouse models of S1PL deficiency and compared the *in vivo* immunological and pathological effects associated with different levels of S1PL enzyme activity. Our findings show that effects on the immune system, typically characterized by lymphopenia and alterations in lymphoid tissues, were present in all mice with reduced or absent S1PL activity. These results indicate that the immune system is particularly sensitive to alterations in S1PL activity. In contrast, the storage disease phenotype in knockout mice that was characterized by alveolar proteinosis, cardiomyopathy, osteopetrosis and urothelial vacuolization in KO mice was prevented by restoration of even low level S1PL activity in humanized mice. In summary, our findings suggest that partial inhibition of S1PL activity can produce potentially therapeutic immunosuppression without causing S1P-related storage disease lesions in non-lymphoid target organs.

## Results

### S1PL is required for normal development in mice

**Figure 1:**
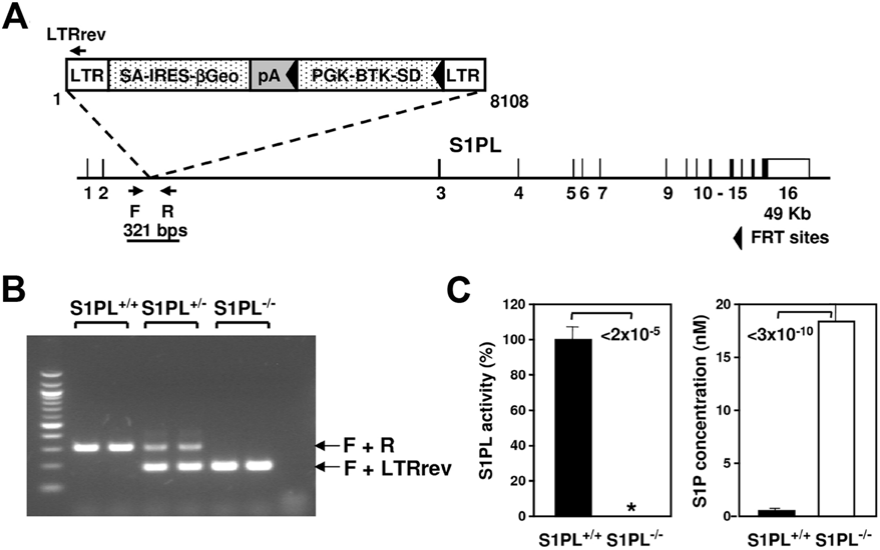
Generation of S1PL mutant mice. (A) Gene trap mutation of the S1PL gene. The insertion occurred within intron 2 of the S1PL gene (NM_009163). LTR, viral long terminal repeat; SA, splice acceptor sequence; IRES, internal ribosome entry site; βGeo, fusion of beta-galactosidase and neomycin phosphotransferase genes; pA, polyadenylation sequence; PGK, phosphoglycerate kinase-1 promoter; BTK-SD, Bruton's tyrosine kinase splice donor sequence. (B) Genotyping strategy. Primers F (5′-TGTAGCAGGCTTTCTTAACTCTGG-3′) and R (5′-TTGGGAAGGTCCTGGTCATTACT-3′) flank the genomic insertion site and amplify a product of 321 nucleotides representing the Wt allele. The LTRrev primer (5′-ATAAACCCTCTTGCAGTTGCATC-3′), complementary to the gene trapping vector, amplifies a 193 nucleotide mutant allele in conjunction with primer F. (C) S1PL activity and S1P concentration were measured in spleen samples of the indicated mice. Enzyme activity was normalized to the mean of the Wt values; 100% corresponds to 172,898±15,344 cpm/mg/hr. Data were pooled from 2 experiments using 4–8 mice of each genotype, and data are presented as mean±SEM. * Activity was below the sensitivity of detection.

S1PL-null mice were derived from an OmniBank [41] embryonic stem cell clone (OST58278) carrying a gene trap mutation within the second intron of the S1PL gene (Fig. 1A and B) Heterozygous mice (S1PL^+/−^) were viable and fertile and were intercrossed to obtain homozygous progeny. Over 900 offspring were produced in an essentially normal (1∶2∶1) Mendelian distribution (26.3% wild type (Wt; S1PL^+/+^), 52.1% S1PL^+/−^, 21.6% S1PL^−/−^), suggesting that S1PL is not essential for embryonic development and neonatal viability. S1PL activity was undetectable in S1PL^−/−^ mice, and was accompanied by elevated levels of S1P in both the spleen (Fig. 1C) and serum (40-fold increase in KO mice compared to Wt). The S1PL^−/−^ mice were smaller than their Wt littermates and failed to thrive during the first weeks of life (bodyweight 15.5±0.7 g, S1PL^+/+^, n = 23 versus 10.3±0.4 g, S1PL^−/−^, n = 20, at 4 weeks of age, p = 3×10^−7^). All S1PL^−/−^ mice in our study died by 15 weeks of age, having a median life span of 29 days (min. 22 days, max. 105 days, n = 55). The reduced viability and blood phenotype (described later) of this S1PL^−/−^ gene-trap line was recapitulated in another S1PL knockout mouse line generated by homologous recombination in which exons 7–8 were replaced by a cre-lox selection cassette (data not shown).

### S1PL^−/−^ mice are lymphopenic due to altered lymphocyte development and tissue egress

**Figure 2:**
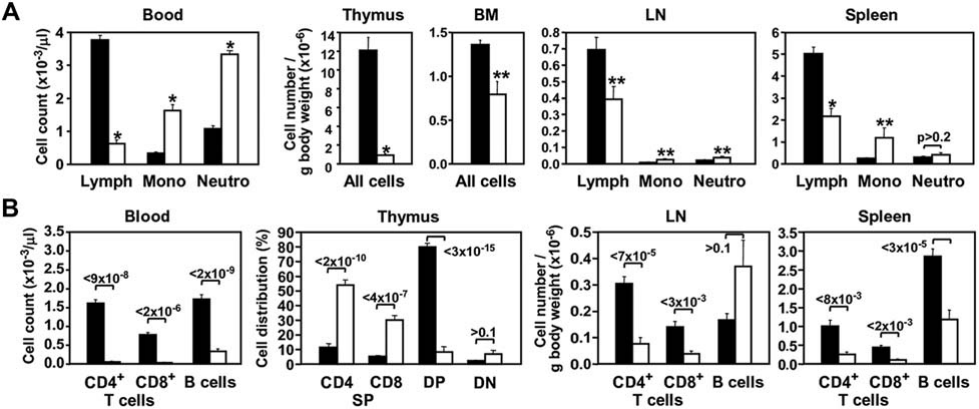
KO of S1PL disrupts both development and recirculation of lymphocytes. (A and B) Hematopoietic cell profile of the indicated tissues from S1PL^+/+^ (black bars) and S1PL^−/−^ (white bars) mice determined by CBC and FACS analysis. Data are presented as mean±SEM values obtained from 11–18 mice of each genotype pooled from two independent experiments giving similar results. Numbers above bars indicate *P* values, *P<2×10^−4^, **P<0.05, S1PL^−/−^ *vs.* S1PL^+/+^. Lymph, lymphocytes; Mono, monocytes; Neutro, neutrophil granulocytes.

Complete blood cell count (CBC) and flow cytometry (FACS) analysis of peripheral blood of 4 week old S1PL^−/−^ mice revealed an almost complete absence of peripheral T cells as well as severely reduced numbers of B cells compared to Wt values (Fig. 2A and B). Despite the severe lymphopenia, the total white blood cell counts of S1PL^−/−^ mice were within the normal range due to a significant increase in monocyte and neutrophil numbers. Likewise, red blood cell counts in the S1PL−/− mice were not significantly different than those of Wt littermates (Wt 8.2+/−0.38, Hom 7.4+/−0.37 p = 0.12). Analysis of lymphoid tissues from S1PL^−/−^ mice demonstrated decreased size and cellularity when cell counts were normalized to body weight (Fig. 2A). The thymus of S1PL^−/−^ animals showed the most pronounced lymphocyte depletion, harboring only approximately 10% the number of lymphocytes seen in S1PL^+/+^ mice. The total lymphocyte numbers in S1PL^−/−^ lymph nodes and spleens were 57% and 43% of those observed in Wt littermates, respectively.

**Figure 3:**
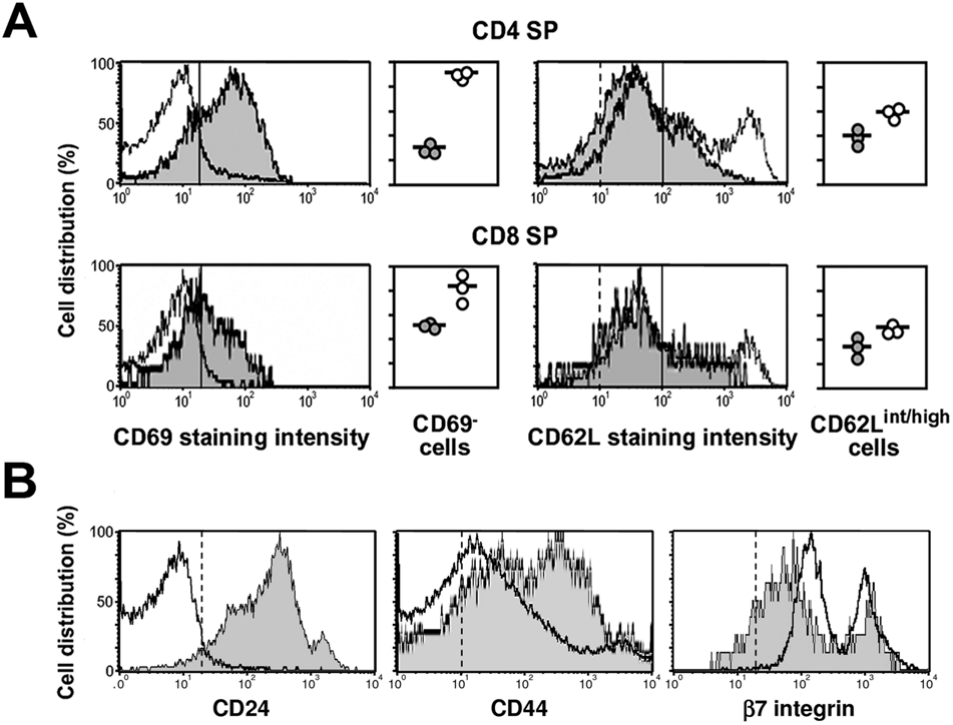
Absence of S1PL leads to accumulation of mature medullary thymocytes. (A) Representative FACS histograms of expression of the CD69 and CD62L markers on S1PL^+/+^ (shaded histograms) and S1PL^−/−^ (open histograms) CD4 and CD8 SP thymocytes. The fraction of electronically gated CD4 and CD8 SP thymocytes that exhibit the indicated phenotypes (demarcated by the solid vertical lines) are depicted in graphical form. Datapoints represent values from individual S1PL^+/+^ (grey circles) and S1PL^−/−^ (white circles) mice. Vertical dashed lines on the CD62L histograms indicate the cut-off values for positive staining obtained with isotype-matched control antibodies, which overlapped with the solid lines on the CD69 histograms. (B) Representative FACS histograms of CD24, CD44, and β7 integrin density on S1PL^+/+^ (shaded histograms) and S1PL^−/−^ (open histograms) SP thymocytes, gated for single expression of either the CD4 or CD8 markers. Vertical dashed lines represent cut-off values as in (A). Samples from two additional mice of each genotype gave similar results.

The altered lymphocyte counts in S1PL^−/−^ mice were similar to those reported following modulation of the S1P-S1P1 receptor axis [28], [31], [34], [38], [39], [42]. Therefore, we assessed the phenotypes of lymphocyte subpopulations residing in the immune tissues by using markers of cell differentiation and lymphocyte egress, such as CD69 and a series of cell adhesion molecules (Fig. 2 and 3). The thymus of S1PL^−/−^ mice showed a severe depletion of CD4 and CD8 double positive (DP) thymocytes compared to controls, but we did not observe an increase in the CD4 and CD8 double negative (DN) cell population as would be expected from a block at the DN to DP transition stage (Fig. 2B). Instead, the proportion of mature CD4 and CD8 single positive (SP) cells was 5-fold higher in the age-matched S1PL^−/−^ mice compared to Wt littermates. The S1PL^−/−^ mature thymocytes had normal surface density of the TCR and the coupled CD3 molecule (data not shown), but completely lacked the expression of CD69, a marker of early medullary thymocytes, which constitute the majority of SP thymocytes in normal controls (Fig. 3A). The S1PL^−/−^ SP thymocytes also showed relatively high expression of CD62L and β7 integrin, but reduced levels of CD24 and CD44 molecules (Fig. 3B). These phenotypic changes in the S1PL^−/−^ mature thymocyte population are consistent with a block in the migration of lymphocytes into the periphery and a concomitant accumulation of mature thymocytes in the thymus of the S1PL-deficient animals [28], [38], [39], [42], [43].

**Figure 4:**
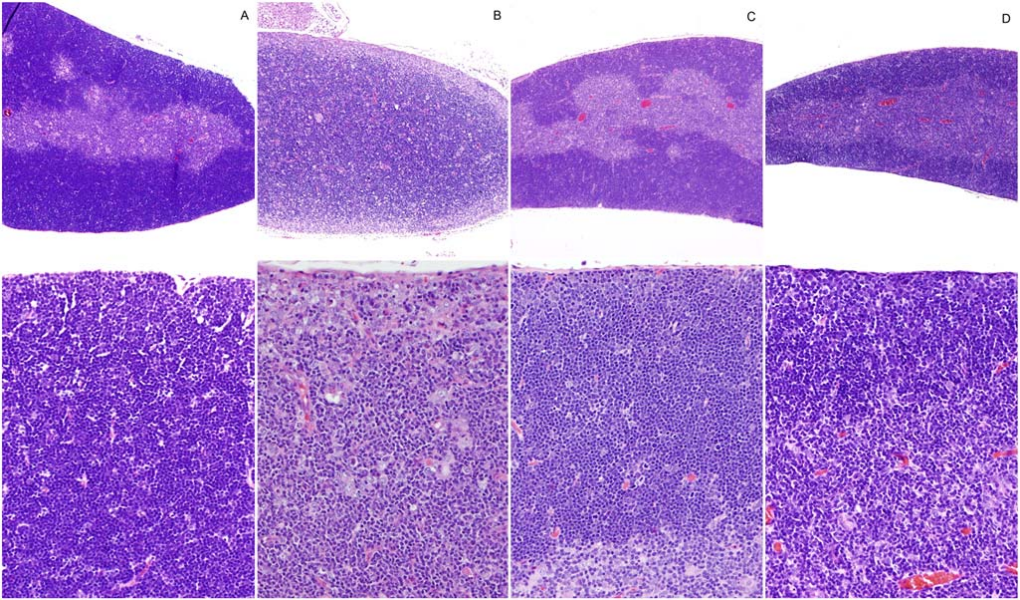
Histopathology of Thymus. (A) S1PL^+/+^ thymus: A broad thymic cortex is densely packed with thymic lymphocytes (lower panel) (B) S1PL^−/−^ thymus: The hypoplastic cortex is very thin and hypocellular due to severe cortical lymphoid depletion and apoptosis. At higher magnification (lower panel) the remaining cells in the thymic cortex include vacuolated stromal/epithelial cells and smaller numbers of granulocytes, macrophages, lymphocytes, and apoptotic cells. In contrast, the markedly expanded and hypercellular thymic medulla of S1PL^−/−^ mice contains numerous mature lymphocytes as well as vacuolated stromal/epithelial cells, macrophages, and histiocytes (lower panel). (C) S1PL^H/H^ thymus: The cortex contains minimally decreased numbers of lymphocytes while there is a minimal expansion of the medulla by increased numbers of lymphocytes (D) S1PL^H/−^ thymus: Thinning of the thymic cortex is accompanied by mild expansion of the medulla due primarily to increased numbers of lymphocytes (lower panel). H&E stain.

Histopathological analysis of thymus from S1PL^−/−^ mice revealed severe cortical lymphoid depletion with concurrent medullary hypercellularity. The thymic cortex of normal age-matched S1PL^+/+^ mice was densely packed with thymic lymphocytes and thus appeared to be broader and more cellular than the thymic medulla (Fig. 4A). This arrangement was essentially reversed in the thymus of S1PL^−/−^ mice, where the hypoplastic thymic cortex appeared as a thin pale hypocellular zone due to the severe, diffuse depletion of thymic lymphocytes (Fig. 4B, upper panel); the few remaining cells in the thymic cortex consisted of vacuolated stromal/epithelial cells mixed with smaller numbers of granulocytes, macrophages, lymphocytes, and apoptotic cells. In contrast, the markedly expanded and hypercellular thymic medulla of S1PL^−/−^ mice contained numerous mature lymphocytes as well as vacuolated stromal/epithelial cells, macrophages, and histiocytes (Fig. 4B, lower panel).

**Figure 5:**
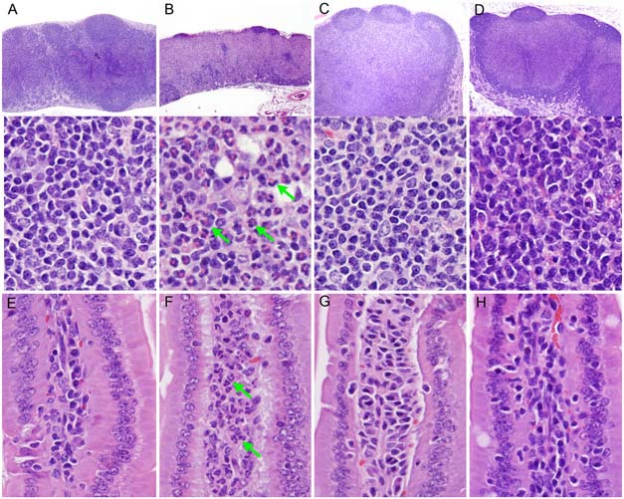
Histopathology of mesenteric lymph nodes and intestinal lamina propria. (A) S1PL^+/+^ lymph node: Paracortical zones are filled primarily with lymphocytes (B) S1PL^−/−^ lymph node: Small B cell lymphoid follicles appear as small semicircular caps overlying an expanded but relatively hypocellular paracortical zones. At higher magnification (lower panel), the paracortex contains markedly increased numbers of granulocytes and macrophages (some vacuolated and containing karyorrhectic debris), mixed with stromal cells and plasma cells. (C) S1PL^H/H^ lymph node: There is marked expansion of paracortical zones. At higher expansion, this expansion is due primarily to sequestered lymphocytes. (D) S1PL^H/−^ lymph node: Similarly, the marked expansion of paracortical zones is due primarily to sequestered lymphocytes (lower panel). (E) S1PL^+/+^ lamina propria: Lymphocytes and plasma cells predominate in the lamina propria of Wt mice. (F) S1PL^−/−^ lamina propria: There is a marked depletion of lymphocytes and plasma cells and increased numbers of granulocytes (arrows) in the intestinal lamina propria. (G) S1PL^H/H^ lamina propria: Essentially normal cell populations are present. (H) S1PL^H/−^ lamina propria: Essentially normal cell populations are present. H&E stain.

**Figure 6:**
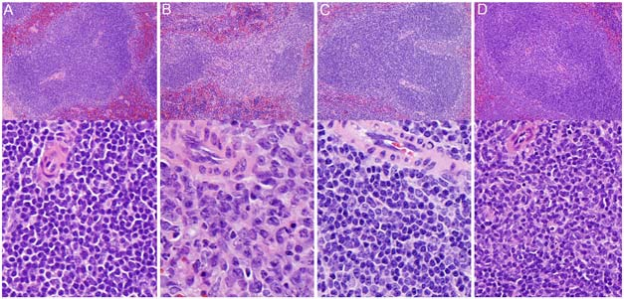
Histopathology of Spleen. (A) S1PL^+/+^ spleen: Densely packed lymphocytes comprise the thick periarteriolar sheaths (PALS) in Wt mice. (B) S1PL^−/−^ spleen: There is severe depletion of T cells in the PALS. The lymphocytes in the PALS have been replaced by small numbers of macrophages, granulocytes, and plasma cells. B cell areas are still evident, with remaining lymphoid follicles and some germinal centers appearing more prominent due to lymphoid depletion of PALS. (C) S1PL^H/H^ spleen: The splenic white pulp contains essentially normal numbers and distributions of lymphocytes in the PALS and follicles. (D) S1PL^H/−^ spleen: Again, the splenic white pulp contains essentially normal numbers and distributions of lymphocytes in the PALS and follicles. H&E stain.

FACS analysis of cell suspensions from the secondary lymphoid organs revealed that loss of S1PL had more consistent effects on T-cell content than B-cell content of these tissues. S1PL^−/−^ mice registered only 25% of normal T-lymphocyte counts in the lymph nodes and spleen. Although the B-cell content was reduced to 41% of the Wt level in the spleen, it was not significantly different in the lymph nodes (Fig. 2B). Histological analysis of S1PL^−/−^ lymph nodes and intestinal mucosa showed that cell types and distributions differed from Wt mouse tissues (Fig. 5A). The S1PL^−/−^ lymph nodes contained smaller B cell follicles and reduced numbers of lymphocytes in paracortical zones (Fig. 5B, upper panel). In mesenteric lymph nodes, lymphoid follicles appeared as small semicircular caps overlying expanded but relatively hypocellular paracortical zones (Fig. 5B, upper panel). The paracortex contained reduced numbers of lymphocytes, but markedly increased numbers of granulocytes and macrophages (some vacuolated and containing karyorrhectic debris), mixed with stromal cells and plasma cells (Fig. 5B, middle panel). The paracortex also showed a mild increase in collagenous stroma. The reduced numbers of lymphocytes and increased numbers of granulocytes in the paracortical areas of the mesenteric lymph nodes were mirrored by depletion of lymphocytes and increased numbers of granulocytes in the lamina propria of the small intestine (Fig. 5B, lower panel). In the spleen, the normal arrangement of periarteriolar lymphoid sheaths (PALS) and lymphoid follicles in the white pulp of Wt (Fig. 6A) was disrupted in S1PL^−/−^ mice (Fig. 6B). The most notable finding was severe depletion of T cells in the PALS in the S1PL^−/−^ spleen (Fig. 6B, lower panel). In contrast, B cell areas were relatively unaffected, with remaining lymphoid follicles and some germinal centers appearing more prominent due to the depletion of PALS. Other findings in the spleen included occasional clusters of plasma cells in the PALS and increased erythroid hematopoiesis in red pulp of S1PL^−/−^ mice.

**Figure 7:**
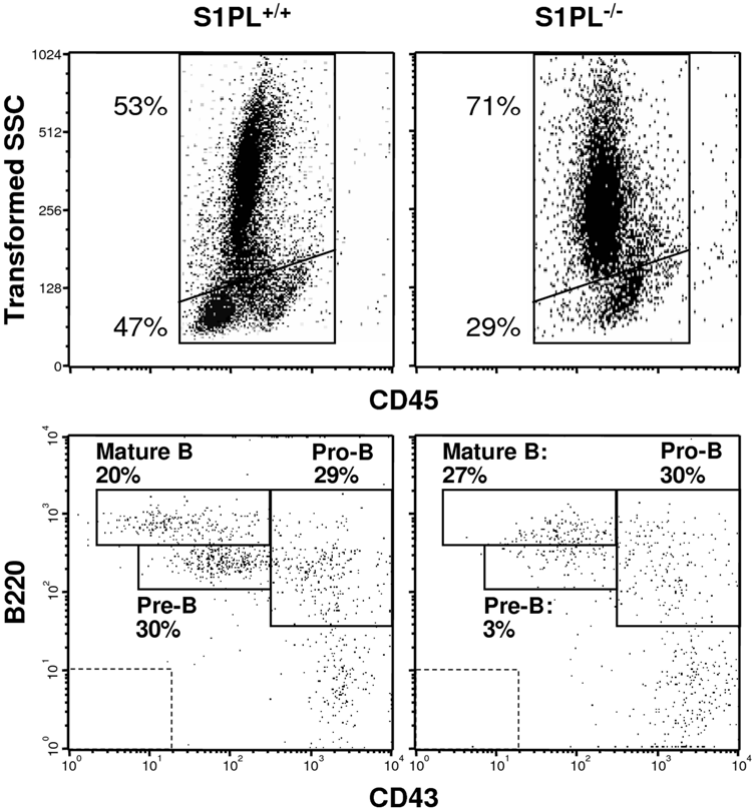
KO of S1PL disrupts hematopoietic cell development in the bone marrow. Representative FACS plots of bone marrow cells from the indicated mice, showing the scatter distribution of CD45 positive leukocytes and the α-B220/CD43 antibody staining patterns of the mononuclear cell fractions. Frequencies of events representing granulocytes, mononuclear cells and the B cell maturation stages are listed close to the respective gates. Dashed rectangles indicate the cut-off values for positive staining obtained with isotype-matched control antibodies. Samples from two additional mice of each genotype gave similar results.

In the bone marrow, FACS analysis showed a lower ratio of mononuclear cells to granulocytes in S1PL^−/−^ compared to S1PL^+/+^ mice as defined by the distinguishing scatter parameters (Fig. 7) and expression of the cell surface marker GR-1 (data not shown). In addition, the fraction of pre-B cells was significantly reduced in the S1PL^−/−^ bone marrow, while the pro-B cell and mature B-cell subsets were present at their usual frequency (Fig. 7). Histopathological examination also showed increased granulocytopoiesis in the bone marrow of S1PL^−/−^ mice (data nor shown).

In summary, comparative assessment of the peripheral blood and primary and secondary lymphoid tissues indicated that the severe lymphopenia in S1PL^−/−^ mice was due to alterations in the development and recirculation of lymphocytes, with the greatest impact on T-cell differentiation and egress.

### Effects of complete S1PL deficiency on non-lymphoid tissues

**Figure 8:**
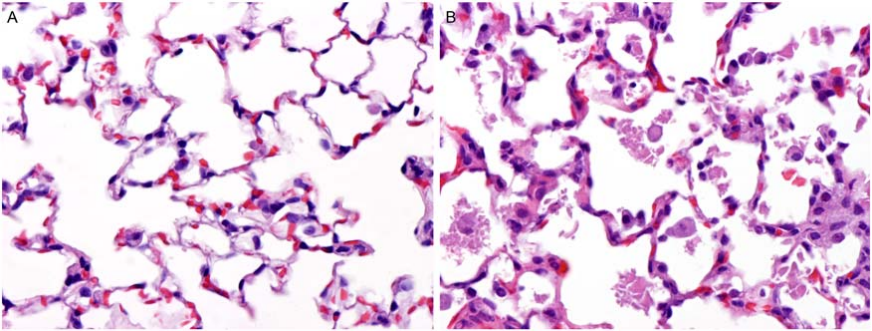
Histopathology of Lung. (A) S1PL^+/+^ lung: Alveolar spaces are generally clear and septa are thin in Wt mice (B) S1PL^−/−^ lung: Alveoli contain variably sized irregularly shaped polygonal flakes and clumps of smooth homogenous (hyaline) material accompanied by increased numbers of alveolar macrophages. Alveolar septa are mildly thickened due to cellular hypertrophy and collagen deposition. H&E stain.

Histopathology of lungs from S1PL^−/−^ mice revealed that virtually all pulmonary alveoli contained variable amounts of variably sized irregularly shaped polygonal flakes and clumps of smooth homogenous (hyaline) material (Fig. 8B). This proteinaceous material was generally free in the lumen but in many locations formed a thin layer lining the surface of alveolar septa. Associated with this alveolar exudate were markedly increased numbers of alveolar macrophages that were often distended by an abundant foamy and microvacuolated cytoplasm. Diffusely, alveolar septa were also mildly thickened due to cellular hypertrophy and collagen deposition.

**Figure 9:**
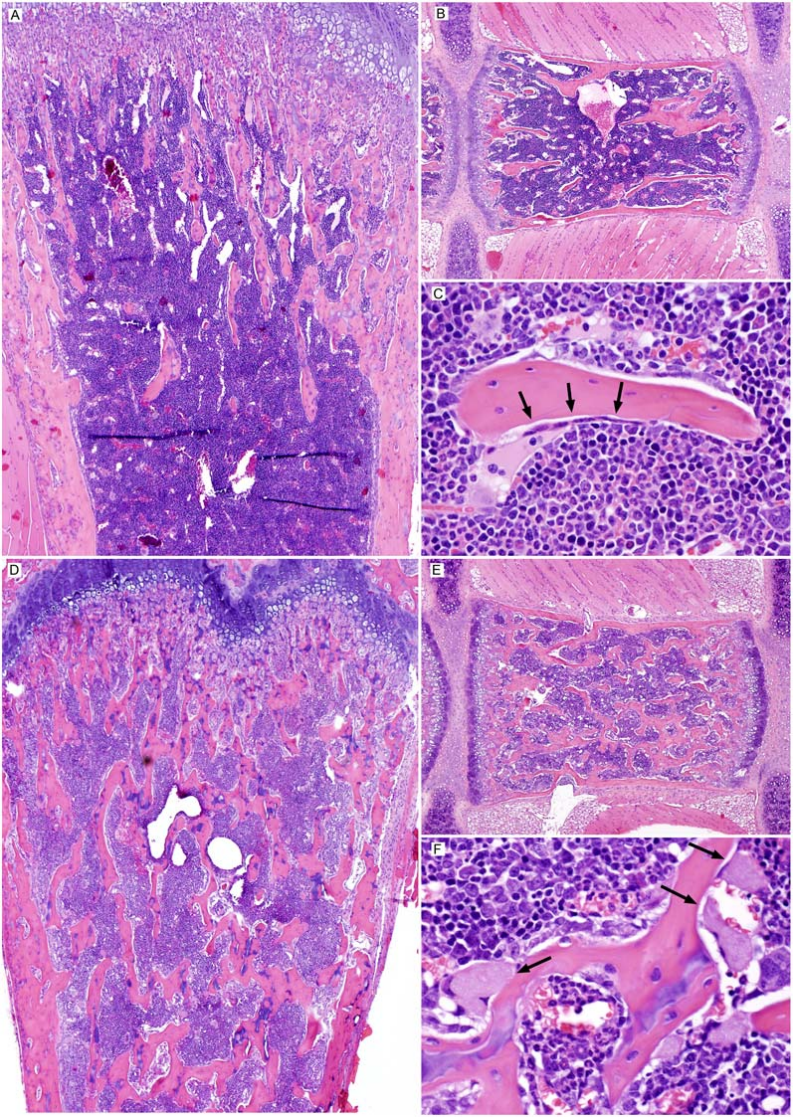
Histopathology of Bone. S1PL^+/+^ Bone (A, B, and C): In Wt mice, the majority of the marrow cavity is filled with hematopoietic elements. Widely spaced trabeculae in long bones (A) and sternum (B) extend a relatively short distance from the physeal growth plate. At higher magnification (C), normal osteoclasts appear as multinucleated cells that are usually tightly adherent and flattened against bone surfaces. S1PL^−/−^ Bone (D, E, and F): In marked contrast, there is a diffuse increase in the volume and extent of trabecular bone in the long bones (D) and sternum (E) S1PL^−/−^ mice. The bony trabeculae extend throughout the marrow farther than normal into the diaphysis of long bones (D) and sternum (E). At higher magnification (F), the more numerous osteoclasts of S1PL^−/−^ mice are distended by abundant pale and granular eosinophilic cytoplasm that displaces cell nuclei to the periphery. These large rounded S1PL^−/−^ osteoblasts (arrows) have reduced direct contact with bone surfaces. H&E stain.

Histological analysis of bone revealed an increase in the volume and extent of trabecular bone in the sternebra and long bones of S1PL^−/−^ mutants. In comparison the bones of Wt mice (Fig. 9, upper panels) bony trabeculae extended throughout the marrow space in sternebrae and farther than normal into the diaphysis in long bones (Fig. 9, lower panels). In contrast to normal osteoclasts, which are usually tightly adherent and flattened against bone surfaces (Fig. 9 upper panels), the more numerous osteoclasts of S1PL^−/−^ mice were distended by an abundant pale and granular eosinophilic cytoplasm that displaced cell nuclei to the periphery (Fig. 9, lower panels). Generally, these large rounded S1PL^−/−^ osteoblasts had minimal direct contact with bone surfaces. They were present in increased numbers around mature trabecular bone in all areas, but they were particularly large and numerous in epiphyseal and diaphyseal regions distant from the physeal growth plate.

**Figure 10:**
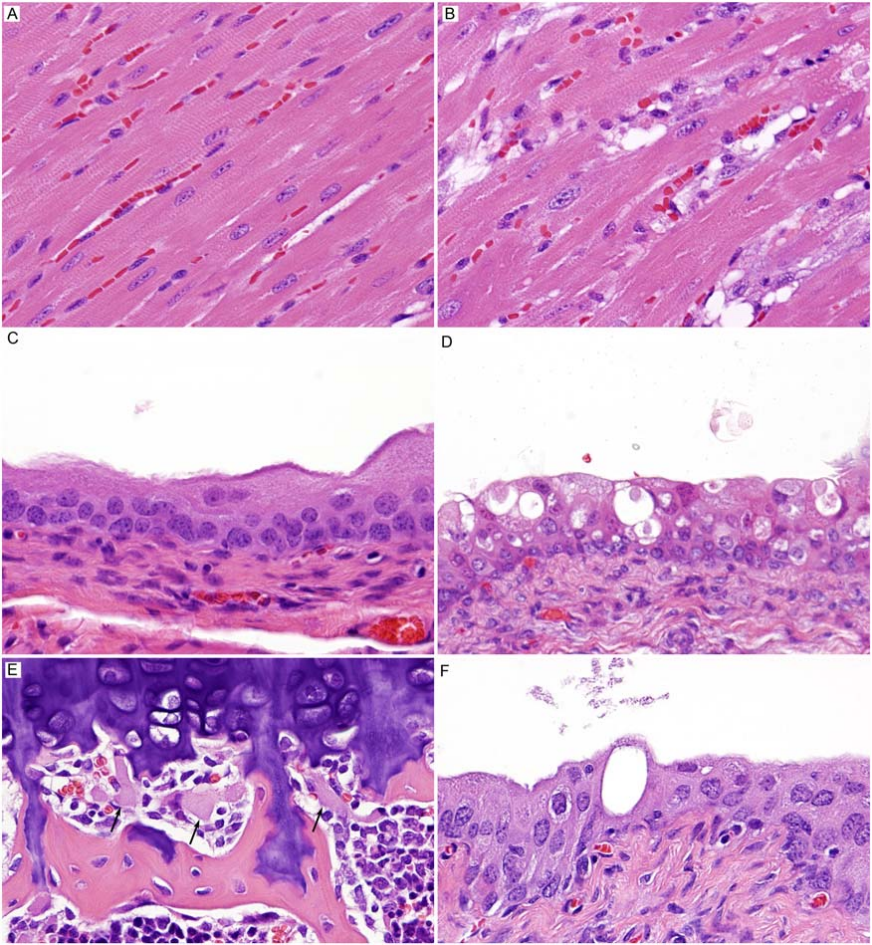
Histopathology in other tissues. (A) S1PL^+/+^ heart: There is minimal interstitial tissue in Wt myocardium. (B) S1PL^−/−^ heart: There is a patchy to diffuse expansion of the interstitium by numerous variably-sized vacuoles and vacuolated mesenchymal cells that separate the cardiomyocytes. Scattered cardiomyocytes contain indistinct foamy inclusions and sharply demarcated clear vacuoles. (C) S1PL^+/+^ urinary bladder: Normal urothelium lines the urinary bladder in Wt mice (D) S1PL^−/−^ urinary bladder: There is widespread ballooning vacuolization, degeneration, and apoptosis of urothelial cell. Lesions are more severe in the superficial umbrella cell layer, where degenerating cells are interspersed among vacuolated urothelial cells. (E) Bone, aged S1PL^H/−^ mouse: Small numbers of distended osteoclasts are present multifocally at the growth plate only. No increase in trabecular bone was evident. (F) Urinary bladder, aged S1PL^H/−^ mouse: Rare vacuolated urothelial cells were present in the superficial mucosa. H&E stain.

Histopathologic lesions in the hearts of S1PL^−/−^ mice were characterized by patchy to diffuse expansion of the interstitium by numerous variably-sized vacuoles and vacuolated mesenchymal cells that separated the cardiomyocytes (Fig. 10B). Scattered cardiomyocytes contained focal cytoplasmic lesions that ranged from indistinct foamy inclusions to sharply demarcated clear vacuoles and some cardiomyocytes had enlarged vesicular nuclei.

S1PL^−/−^ mice also developed lesions affecting the urothelium lining the renal pelvis, ureters, and urinary bladder. These urothelial lesions were characterized by widespread ballooning vacuolization, degeneration, and apoptosis of urothelial cells (Fig. 10D). The most severe lesions were present in the superficial umbrella cell layer of urinary bladder urothelium, where small numbers of degenerating cells were interspersed among urothelial cells distended by large vacuoles that were either empty or partially filled with pale eosinophilic granular material.

### Humanized knock-in mice are lymphopenic but do not develop non-lymphoid pathologies

**Figure 11:**
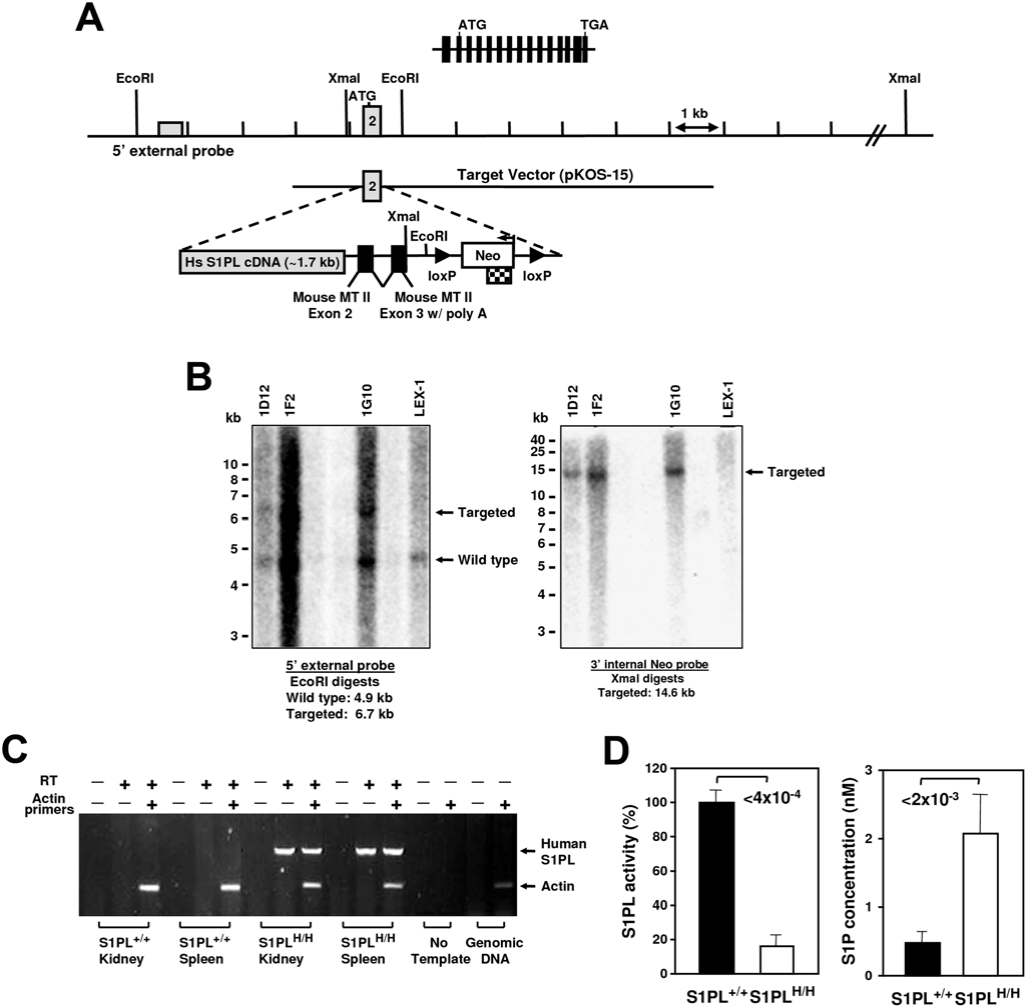
Generation of humanized S1PL allele. (A) Schematic representation of the S1PL allele. Entire genomic locus spanning 15 exons indicated at the top. To create the humanized line, the coding portion of mouse exon 2 (first coding exon) was deleted and replaced with a full length human S1PL cDNA along with a LoxP-flanked *Neo* marker and a minigene to allow splicing. Hs, *Homo sapiens*; MT II, *metallothionein 2* gene; Neo, neomycin resistance gene. (B) Genotyping strategy. Three correctly targeted clones (1D12, 1F2, 1G10) were identified by Southern blot analysis using 5′ external and 3′ internal probes. Lex-1, non-targeted, control ES cell DNA. (C) Expression analysis. Expression of the human S1PL gene was detected by RT-PCR. (D) S1PL activity and S1P concentration were measured in spleen samples of the indicated mice. Data are presented as in Fig. 1C.

**Figure 12:**
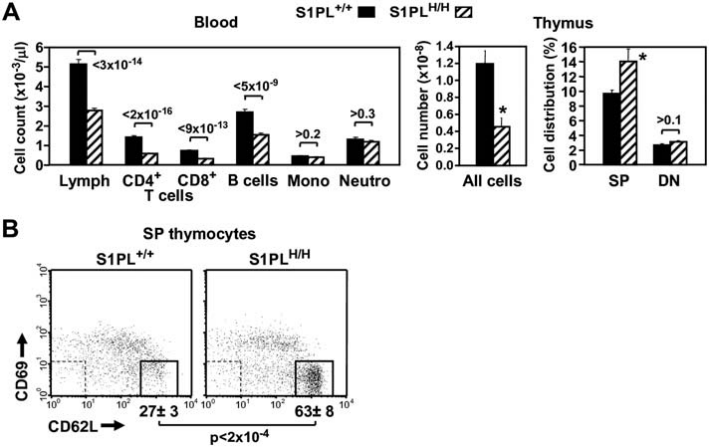
Human S1PL knock-in results in partial rescue of KO hematopoietic cell profile. (A) Blood cell counts were obtained from 62–75 mice of each genotype pooled from seven independent experiments giving similar results. Total thymocyte counts and fractions of SP and DN cells were determined from 3 and 15 mice of each genotype, respectively. Data are presented as described in Fig. 2A and B; *P<0.02, S1PL*^H/H^ vs.* S1PL^+/+^. (B) Representative FACS plots show the CD69/CD62L Ab staining patterns of SP thymocytes from the indicated mice, identified by single expression of either the CD4 or CD8 markers. Data are presented as described in Fig. 7. Samples from 11 additional mice of each treatment group gave similar results.

In order to obtain a mouse model for testing *in vivo* effects of S1PL-selective compounds and to rescue the defects observed in S1PL^−/−^ mice we generated mice that express a human transgene. For this purpose, human S1PL cDNA was inserted at the mouse S1PL locus by homologous recombination, replacing the first exon of the mouse gene (Fig. 11). The resulting knock-in S1PL^H/H^ mice exhibited S1PL activity at approximately 17% of normal Wt levels, which correlated with increased levels of S1P in the spleen (Fig. 11D) and serum (4-fold increase in humanized mice compared to Wt). The limited S1PL activity in the humanized S1PL^H/H^ mice alleviated the pathological findings observed in the lungs, heart, bone, and urinary tracts of S1PL^−/−^ mice, but did not rescue completely the immunological phenotypes. Transgenic S1PL^H/H^ mice were viable and fertile, but registered significantly reduced peripheral lymphocyte numbers compared to Wt littermates (Fig. 12A). RBC numbers in the humanized mice were indistinguishable from Wt (Wt 10.0+/−0.16, S1PLH/H 10.0+/−0.19). T-cell counts in S1PL^H/H^ mice were decreased substantially (by about 60%) in the peripheral blood and thymus, although not in the secondary lymphoid organs (Fig. 12B). Similarly, reduced B-cell numbers (by about 40%) were observed only in the peripheral blood, while monocyte and granulocyte counts were normal. FACS analysis of the thymus of S1PL^H/H^ mice showed the characteristic increase in the proportion of single positive thymocytes (Fig. 12A) with markers of recent thymic emigrants (Fig. 12B) also being observed in S1PL^−/−^ mice.

A histological analysis of S1PL^H/H^ animals at 16 weeks of age revealed relatively mild lesions that were restricted to lymphoid tissues and consistent with retention of mature lymphocytes in the thymus and secondary lymphoid organs. None of the lesions seen in S1PL^−/−^ mice were noted in the lung, heart, bone, or urothelium of young adult S1PL^H/H^ mice. In the thymus, the cortex contained minimally decreased numbers of lymphocytes (Fig. 4C upper panel) accompanied by a minimal expansion of the medulla by increased numbers of lymphocytes (Fig. 4C lower panel). The mesenteric and peripheral lymph nodes of S1PL^H/H^ mice were characterized by marked expansion of paracortical areas by sequestered lymphocytes (Fig. 5C). In contrast to the severe depletion of lymphocytes in the PALS of S1PL^−/−^ mice (Fig 6B), the splenic white pulp of S1PL^H/H^ mice (Fig. 6C) contained essentially normal numbers and distributions of lymphocytes in the PALS and follicles. In aged (58 weeks) S1PL^H/H^ mice, notable histopathological changes were also limited to lymphoid tissues, although there was minimal vacuolization of a few superficial urothelial cells in the urinary bladder of one of the six aged humanized mice examined (data not shown).

**Figure 13:**
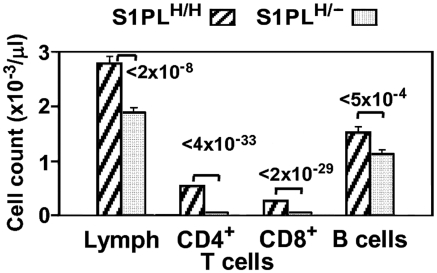
Lymphopenia correlates proportionally with the extent of S1PL deficiency. Blood cell counts were obtained from 75 and 30 mice carrying 2 copies or a single copy of the human knockin allele, respectively. Data are presented as described in Fig. 12A.

To assess the phenotypic effects of even greater decreases in S1PL activity, we crossed S1PL^H/H^ with S1PL^−/−^ mice to obtain S1PL^H/−^ offspring which carry a single functional copy of S1PL. S1PL activity in these S1PL^H/−^ mice was expected to be lower than the enzyme activity in S1PL^H/H^ mice. In fact, S1PL activity was below the level of detection levels of our enzymatic assay (data not shown). These lower levels of S1PL activity in S1PL^H/−^ mice correlated with an additional significant reduction in circulating T lymphocytes compared to S1PL^H/H^ animals (Fig. 13). Histopathological changes in lymphoid tissues in S1PL^H/−^ mice were similar in type and severity to those seen in the S1PL^H/H^ line (Fig. 4D, Fig. 5D, and Fig. 6D). Importantly, the additional reduction of S1PL activity in the S1PL^H/−^ mice still did not elicit clinically significant lesions in any non-lymphoid tissues of young adult mice. Similarly, aged (58 weeks) S1PL^H/−^ mice also lacked lesions in lungs or hearts, although small numbers of enlarged osteoclasts at the physis in bones (Fig. 10E), and multifocal minimal vacuolization and degeneration of urinary bladder urothelium (Fig. 10F), were present. Nevertheless, the amounts and distribution of trabecular bone in the metaphysis and diaphysis of long bones were within normal limits in aged S1PL^H/−^ mice.

## Discussion

Comparative analysis of KO and humanized mice demonstrated differential effects of complete and partial inhibition of S1PL activity on general physiology versus immune function. Inactivation of S1PL resulted in increased levels of S1P in tissues and serum, and the ensuing physiological consequences correlated proportionally with the extent of S1PL deficiency. We found that clinically significant lesions in non-lymphoid tissues developed only in the complete absence of S1PL activity in the S1PL^−/−^ mice. In contrast, the immune system was exquisitely sensitive to alterations in S1PL activity even in partial absence of S1PL. Concurrent depletion of T cells from the peripheral blood and sequestration of mature T cells within the thymic medulla and lymph nodes were observed in all three genetic models (S1PL^−/−^, S1PL^H/H^, and S1PL^H/−^ mice). The T-cell content of secondary lymphoid organs was always less affected than would be expected from the severity of lymphopenia in the peripheral blood, even after complete loss of S1PL activity. Such altered redistribution of T lymphocytes is a hallmark of modulation of the S1P-S1P1 receptor axis and can be explained by accumulation of S1P in tissues of our S1PL-deficient genetic models. Our results are consistent with published data showing that RNA interference–mediated knockdown of S1PL in hematopoietic cells leads to increased S1P levels and a block in T-cell egress [28]. In the same study, inhibition of S1PL by the food colorant THI produced similar results [28]. Our analysis of genetic models with a wide range of systemic S1PL deficiency extend on these studies and clearly demonstrate that T-cell trafficking is particularly tightly controlled by S1PL activity compared to other physiological processes.

It appears that several mechanisms may be involved in S1PL-induced inhibition of thymocyte egress and development. It is a novel finding in our genetic models that excessive S1P levels can also disturb thymocyte development in addition to T-cell egress, leading to decreased thymic cellularity and T-cell output. This is most clearly evidenced in S1PL^−/−^ mice by the severe hypocellularity and increased apoptosis of lymphocytes within the thymic cortex, and by the paucity of T-cells in the splenic PALS and paracortical areas of lymph nodes. It is possible that stress-related elevations in glucocorticoid levels could have contributed to lymphocyte apoptosis and hypocellularity in the thymic cortex of S1PL^−/−^ mice; however, the lymphoid hypercellularity in the thymic medulla of these mice is not generally a feature of stress-induced thymic atrophy, suggesting that direct S1PL effects are involved. Direct S1PL effects on lymphocyte development in the thymus are also suggested by the lymphoid hypocellularity and increased apoptosis of lymphocytes we observed in the thymic cortex of clinically normal S1P ^H/−^ mice; these mice retained low level S1PL activity and the cortical lymphoid depletion was unlikely to be stress related. S1P has been shown to both promote and protect against apoptosis in different mammalian cells [2], [6]–[8], [10]–[13], and the S1P1 receptor-agonist FTY720 affects thymocyte apoptosis in mice and rats [44], [45]. In addition to its direct effect on apoptosis, excess tissue concentrations of S1P may also interfere with lympho-stromal interactions. In S1PL^−/−^ mice, thymic epithelial cells and stromal cells that are required for T-cell selection and maturation, were highly vacuolated, which might compromise their function. The severe vacuolization of epithelial cells in the thymic cortex was largely reversed by low-level expression of S1PL in the humanized mice, but the thymus of these mice still registered a 60% reduction in thymic cellularity. Despite this apparent thymic hypocellularity, no loss of either the progenitor DN T-cell population or the mature T-cell population in the thymus was observed in the humanized mice. This suggests that loss of S1PL activity impedes the intermediary steps of T-cell maturation, which are dependent upon a complex set of lympho-stromal interactions [46]. Furthermore, we observed sequestration of lymphocytes in the thymic medulla and lymph nodes of the humanized mice but not in the spleen. These findings are consistent with some published reports that S1P-mediated retention of lymphocytes is prevalent in the thymus and lymph nodes but not in spleen, which further suggests that a tissue-specific stromal component may be involved in the S1P1 receptor-mediated control of lymphocyte trafficking [25], [38], [46]. It must be noted however that the absence of significant sequestration of lymphocytes in the spleen in our studies is in apparent conflict with other recent reports that provided evidence supporting a role for S1P in egress of both B-cells [32] and T-cells [47] from the spleen.

Our finding that S1PL inhibition had a much lesser impact on B-cell homeostasis than its effects on T lymphocytes is consistent with reported differences in the ability of FTY720 or the S1PL inhibitor THI to retain T- versus B-lymphocytes [28], [30], [32]. We detected B-cell development abnormalities only in the S1PL^−/−^ bone marrow. Similarly, increased myeloid cell numbers in the peripheral blood and infiltration of the peripheral organs was evident only in S1PL^−/−^ mice. One explanation for the increased myeloid cell activity may be that the mobilized neutrophils and phagocytes are involved in eliminating apoptotic cells that accumulate in the infiltrated organs. Similar myeloid cell infiltration was observed in the thymus, spleen and LN of *lpr* mice after treatment with FTY720 which induced apoptosis in these organs [48]. Consistent with this explanation is our finding that neutrophil granulocytes of S1PL^−/−^ mice show a significantly decreased expression of CD62L (our unpublished data), an adhesion molecule which is shed from the cell surface after cell activation, implying that granulocytes are in an activated state in these animals.

In contrast to the failed rescue of immune system phenotypes in our humanized mice, we found that even minimal S1PL activity was sufficient to prevent the development of significant lesions in non-lymphoid tissues. Among the non-lymphoid tissues, the urothelium appeared to be the most sensitive to reduced S1PL activity. Urothelial lesions could be caused by a direct effect of S1PL deficiency in urothelial cells or by excreted sphingolipid metabolites present in the urine. However, urothelial lesions were entirely absent in young adult humanized mice and were minimal in aged S1PL^H/−^ and S1PL^H/H^ mice. Similarly, lung pathology was not present in either of the genetic models expressing partial S1PL activity. Respiratory distress caused by pulmonary lesions was the most likely cause of reduced viability of S1PL^−/−^ mice, as accumulations of proteinaceous exudates lining and partially filling alveoli would impair alveolar gas exchange. It is likely that the extremely elevated levels of extracellular S1P played a direct role in the development of the pulmonary lesions in the complete S1PL knockouts. One of the S1P receptors, S1P3, is expressed on alveolar epithelial cells, and airway administration of S1P has been shown to disrupt epithelial cell tight junctions and induce pulmonary edema [49]. It is likely that S1P levels are elevated in the alveoli of S1PL^−/−^ mice and that activation of alveolar epithelial S1P3 might compromise the integrity of the pulmonary epithelial barrier. In addition, impaired function of the distended alveolar macrophages in lungs of S1PL−/− mice may also have contributed to the accumulation of proteinaceous alveolar exudates. Mutations that directly impair the development and/or function of alveolar macrophages [50], as well as mutations that inhibit degradation of surfactant within these cells, have been shown to result in alveolar proteinosis and pulmonary dysfunction in both mouse and human [51]. Surfactant dysregulation has also been proposed as a common pathogenic mechanism of several sphingolipid storage disorders [52].

The increased interstitial cellularity and vacuolation in the myocardium and osteopetrosis seen with the S1PL^−/−^ mice also resolved with the minimal enzyme reconstitution in the humanized mouse lines. The myocardial lesions, while not clearly degenerative, suggest that myocardial homeostasis was altered. The molecular mechanisms responsible for the bone phenotype in the S1PL^−/−^ mice are unclear at this point since studies have implicated S1P signaling in both osteoclast and osteoblast function [53]. However, S1PL^−/−^ mice had an increased volume of trabecular bone in both sternebra and long bones (osteopetrosis). Bone normally undergoes a constant process of remodeling carried out by osteoclasts which resorb old bone and osteoblasts, generating new bone. The osteopetrosis seen in S1PL^−/−^ mice appears to be the result of dysfunctional osteoclasts. The enlarged osteoclasts in S1PL^−/−^ mice were distended by intracellular accumulations of granular eosinophilic material and were generally detached from the bone surfaces, and would therefore be physically unable to form the tight junctions and ruffled borders required for effective bone resorption.

In summary, we found that loss of S1PL activity in S1PL^−/−^ mice results in severe T-cell depletion in the blood, thymus, spleen, and lymph nodes, as well as lesions in the lung, heart, urinary system, and bone, and that the observed reduced viability of S1PL^−/−^ mice is most likely due to respiratory failure. Partial restoration of S1PL activity in humanized mice rescued lesions in lung, bone, and urothelium, but failed to restore normal T-cell development and trafficking. Thus, comparative analysis of genetic mouse models having differing levels of S1PL activity suggests that the S1P pathway is more stringently controlled in the immune system than in the non-lymphoid environment. The relatively specific physiological response to partial inhibition of S1PL activity suggests that there is a window within which reduced S1PL activity could produce potentially therapeutic immunosuppression without causing clinically significant S1P-related lesions in non-lymphoid target organs. Immunosuppression induced by inhibiting S1PL activity has potential application in the treatment of a variety of autoimmune and inflammatory diseases, and the humanized S1PL mouse line provides an attractive model for studying small molecule inhibitors of the human enzyme.

## Materials and Methods

### Experimental Animals

All mice analyzed were maintained on a mixed genetic background (129S5/SvEvBrd and C57BL/6J) at the ALAAC-accredited animal facility at Lexicon Pharmaceuticals Inc. Studies of gene-disrupted or humanized animals were performed using Wt littermates as controls. Mice were housed in a barrier facility at 22°C on a fixed 12-hour light and 12-hour dark cycle and were fed rodent chow # 5001 (Purina, St. Louis, MO) *ad libitum*. Procedures involving animals were conducted in conformity with the Institutional Animal Care and Use Committee guidelines that are in compliance with the state and federal laws and the standards outlined in the Guide for the Care and Use of Laboratory Animals.

### Generation of S1PL KO mice

Homozygous S1PL-null mice were generated by gene trapping as previously described [41], [54]. OmniBank embryonic stem cell clone OST58278 was selected for microinjection based on sequence similarity to the mouse S1PL gene (accession number NM_009163). The genomic insertion site of the gene trap vector in OST58278 was determined by inverse PCR [54] (Fig. 1A). Mice carrying this mutation were generated by using standard methods of host embryo microinjection of embryonic stem cells, chimera production, and germ-line transmission [54]. Animal genotypes were determined by quantitative PCR [54] (Fig. 1B).

### Generation of human S1PL knockin allele

The first coding exon of the mouse S1PL gene was replaced with the human S1PL cDNA using the Lambda KOS system [55]. The Lambda KOS library was screened by PCR using the following primers: 5′-GCTTTTGCTCAAAGCTCAGC-3′ and 5′-TGTACCTGCTAAGTTCCAGG-3′. A single genomic clone (pKOS-15) was isolated and confirmed by sequence analysis. Gene-specific arms 5′-AGCCCCGGGGAGGGAGCCGGCTGCAGAGGAAG-3′ and 5′-CTCAGAGCCTTCTGAGCGAGAGAGAAAGGA-3′ were appended by PCR to a yeast selection cassette containing the *URA3* marker. The yeast selection cassette and pKOS-15 were co-transformed into yeast, and clones that had undergone homologous recombination to replace a 37 bp region containing the first coding exon of S1PL with the yeast selection cassette were isolated. The yeast cassette was subsequently replaced with a cassette containing a spliceable mini-gene, the human S1PL cDNA and the Neo selectable marker flanked by LoxP sites (Fig. 11A). The linearized targeting vector was electroporated into 129S5/SvEvBrd embryonic stem cells that contain a Protamine-driven *Cre* recombinase transgene to allow excision of Neo in the germline of transgenic animals [56]. RT-PCR analysis was used to demonstrate expression of the human cDNA (Fig. 8B).

### Measurement of S1PL activity

Spleens of mice were homogenized in ice-cold buffer containing 50 mM KPO_4_, pH 7.5, 1 mM EGTA, 1 mM DTT, and complete protease inhibitor cocktail (Roche Applied Science, Indianapolis, IN). The homogenate was centrifuged at 2,500×g for 10 minutes, and the supernatant was used for enzyme activity measurements. Protein concentration was determined using the Bradford method. S1PL activity was determined using ^33^P-labeled S1P as substrate. The reactions were carried out in a 50 µl volume containing 100 mM KPO_4_, pH 7.4, 1 mM EDTA, 25 mM NaF, 5 mM DTT, phosphatase inhibitors I & II (Sigma-Aldrich), 0.1% Triton X-100 and various amounts of tissue lysate. The reactions were initiated by addition of pyridoxal phosphate (final concentration 50 µM) and 15 nCi of [^33^P]-S1P (specific activity 0.6 Ci/mmol, total S1P concentration 0.5 µM). The reaction mixtures were incubated at 37°C for 1 hour and terminated with the addition of 90 µl of 35% acetonitrile containing 0.1% TFA. A total of 120 µl of the mixtures were then transferred to a 96-well C18 multi-SPE plate (Millipore Corporation, Bedford, MA) pre-equilibrated first with 100 µl MeOH and then with 200 µl 20% acetonitrile containing 0.1% TFA. The plate was briefly centrifuged at 3,000 rpm for 1 minute and the filtrate was collected and assayed for ^33^P activity by scintillation counting. These reactions were performed in the presence and absence of 2.5 or 5 mM semicarbazide, a reactive compound that inhibits S1PL activity [57]. Activities reported represent the semicarbazide-sensitive portion of the total activity.

### Measurement of S1P concentration

Extraction of S1P from mouse tissues was performed essentially as described [58] and dried under N_2_. S1P levels in dried extracts were quantitated using a whole cell receptor binding assay [59]. Briefly, S1P extracts or standards were resuspended in 50 µl binding buffer containing 25,000 cpm [^33^P]-S1P (specific activity 0.6 Ci/mmol) and added to 5×10^4^ HEK293 cells expressing S1P1 receptor. Cells were incubated on ice for one hour then washed twice with binding buffer. Cells were lysed by addition of 100 µl lysis buffer and 40 µl of the resulting extract was added to 120 µl MS40 microscint. S1P content of extracts was estimated from standard curves (between 42 pM and 2.5 µM) generated with cold S1P standards.

### CBC and flow cytometry

CBC analysis was performed on blood isolated from the retroorbital sinus of mice anesthetized with isofluorane using either Cell-Dyn 3500 (Abbott Diagnostics, Abbott Park, IL) or HemaVet 850 FS (Drew Scientific, Inc., Oxford, CT) instruments. For flow cytometry analysis of whole blood, erythrocytes were lysed by hypotonic shock, washed once in FACS wash buffer (FWB: PBS/0.1% BSA/0.1% NaN_3_/2 mM EDTA) and stained for 30 minutes at 4°C in the dark with the indicated combination of fluorochrome-conjugated mAbs. After staining, the cells were washed once with FWB and resuspended in 300 µl FWB for analysis. Samples were analyzed using a FACSCalibur flow cytometer and CellQuest Pro software (Becton Dickinson Immunocytometry Systems, San Jose, CA). Thymus, bone marrow (from two femurs per mouse), spleen, and lymph nodes (LN) (two inguinal, axillary, lateral axillary, and mesenteric per mouse) were harvested and dispersed into single-cell suspensions by forcing the tissue through a 70 µm cell strainer (Falcon, Becton Dickinson Labware, Bedford, MA). Single cell suspensions were analyzed by CBC and FACS as above except that prior to staining, cells were incubated with 10 µl antibody (1/10 dilution in FWB, Fc Block®, BD BioSciences, San Jose, CA) for 15 min. at 4°C. The following antibodies (all from BD BioSciences, San Jose, CA) were used in this study: anti-B220-FITC, -PE, -APC and -PERCP, anti-β7 integrin-FITC and -PE, CD4-FITC, -PE, -APC and -PERCP, CD8a- FITC, -PE, -APC and -PERCP, CD69-FITC, CD44-APC, CD24-FITC, -PE and -APC, CD62L-FITC, -PE and –APC, CD43-PE, CD45- FITC, -PE, -APC and –PERCP. The absolute number of each indicated cell subset was calculated by multiplying its fractional representation determined by FACS by the absolute number of cells identified by CBC. B cells were identified as CD19^+^/CD45^+^/TcRβ^−^ mononuclear cells, T cells as CD4^+^ or CD8^+^ and TcRβ^+^/CD45^+^/CD19^−^ mononuclear cells.

### Histopathology

Tissues collected from genetically modified mice and age-matched Wt control mice were immersion fixed in 10% neutral buffered formalin, except eyes, which were fixed for 24 h in Davidson's fixative. All tissues were embedded in paraffin, sectioned at 4 µm, and mounted on positively charged glass slides (Superfrost Plus, Fisher Scientific, Pittsburgh, PA) and stained with hematoxylin and eosin for histopathologic examination.

### Statistics

Analysis of statistical significance of group differences was performed using the two-sample t-test. In all tests, a P value of <0.05 was considered significant.

## References

[1] Chalfant CE, Spiegel S (2005). Sphingosine 1-phosphate and ceramide 1-phosphate: expanding roles in cell signaling.. J Cell Sci.

[2] Hait NC, Oskeritzian CA, Paugh SW, Milstien S, Spiegel S (2006). Sphingosine kinases, sphingosine 1-phosphate, apoptosis and diseases.. Biochim Biophys Acta.

[3] Leclercq TM, Pitson SM (2006). Cellular signalling by sphingosine kinase and sphingosine 1-phosphate.. IUBMB Life.

[4] Pyne S, Pyne NJ (2000). Sphingosine 1-phosphate signalling in mammalian cells.. Biochem J.

[5] Saba JD, Nara F, Bielawska A, Garrett S, Hannun YA (1997). The BST1 gene of Saccharomyces cerevisiae is the sphingosine-1-phosphate lyase.. J Biol Chem.

[6] Goetzl EJ, Kong Y, Mei B (1999). Lysophosphatidic acid and sphingosine 1-phosphate protection of T cells from apoptosis in association with suppression of Bax.. J Immunol.

[7] Hisano N, Yatomi Y, Satoh K, Akimoto S, Mitsumata M (1999). Induction and suppression of endothelial cell apoptosis by sphingolipids: a possible in vitro model for cell-cell interactions between platelets and endothelial cells.. Blood.

[8] Hung WC, Chuang LY (1996). Induction of apoptosis by sphingosine-1-phosphate in human hepatoma cells is associated with enhanced expression of bax gene product.. Biochem Biophys Res Commun.

[9] Liao JJ, Huang MC, Graler M, Huang Y, Qiu H (2007). Distinctive T cell-suppressive signals from nuclearized type 1 sphingosine 1-phosphate G protein-coupled receptors.. J Biol Chem.

[10] Maceyka M, Sankala H, Hait NC, Le Stunff H, Liu H (2005). SphK1 and SphK2, sphingosine kinase isoenzymes with opposing functions in sphingolipid metabolism.. J Biol Chem.

[11] Moore AN, Kampfl AW, Zhao X, Hayes RL, Dash PK (1999). Sphingosine-1-phosphate induces apoptosis of cultured hippocampal neurons that requires protein phosphatases and activator protein-1 complexes.. Neuroscience.

[12] Oskouian B, Sooriyakumaran P, Borowsky AD, Crans A, Dillard-Telm L (2006). Sphingosine-1-phosphate lyase potentiates apoptosis via p53- and p38-dependent pathways and is down-regulated in colon cancer.. Proc Natl Acad Sci U S A.

[13] Reiss U, Oskouian B, Zhou J, Gupta V, Sooriyakumaran P (2004). Sphingosine-phosphate lyase enhances stress-induced ceramide generation and apoptosis.. J Biol Chem.

[14] Saba JD, Hla T (2004). Point-counterpoint of sphingosine 1-phosphate metabolism.. Circ Res.

[15] Cyster JG (2005). Chemokines, sphingosine-1-phosphate, and cell migration in secondary lymphoid organs.. Annu Rev Immunol.

[16] Rosen H, Goetzl EJ (2005). Sphingosine 1-phosphate and its receptors: an autocrine and paracrine network.. Nat Rev Immunol.

[17] Ikeda M, Kihara A, Igarashi Y (2004). Sphingosine-1-phosphate lyase SPL is an endoplasmic reticulum-resident, integral membrane protein with the pyridoxal 5′-phosphate binding domain exposed to the cytosol.. Biochem Biophys Res Commun.

[18] van Veldhoven PP, Mannaerts GP (1993). Sphingosine-phosphate lyase.. Adv Lipid Res.

[19] Ito K, Anada Y, Tani M, Ikeda M, Sano T (2007). Lack of sphingosine 1-phosphate-degrading enzymes in erythrocytes.. Biochem Biophys Res Commun.

[20] Sillence DJ, Platt FM (2003). Storage diseases: new insights into sphingolipid functions.. Trends Cell Biol.

[21] Schmahl J, Raymond CS, Soriano P (2007). PDGF signaling specificity is mediated through multiple immediate early genes.. Nat Genet.

[22] Liao JJ, Huang MC, Goetzl EJ (2007). Cutting edge: Alternative signaling of Th17 cell development by sphingosine 1-phosphate.. J Immunol.

[23] Pettus BJ, Chalfant CE, Hannun YA (2004). Sphingolipids in inflammation: roles and implications.. Curr Mol Med.

[24] McVerry BJ, Garcia JG (2005). In vitro and in vivo modulation of vascular barrier integrity by sphingosine 1-phosphate: mechanistic insights.. Cell Signal.

[25] Sanna MG, Wang SK, Gonzalez-Cabrera PJ, Don A, Marsolais D (2006). Enhancement of capillary leakage and restoration of lymphocyte egress by a chiral S1P1 antagonist in vivo.. Nat Chem Biol.

[26] Wei SH, Rosen H, Matheu MP, Sanna MG, Wang SK (2005). Sphingosine 1-phosphate type 1 receptor agonism inhibits transendothelial migration of medullary T cells to lymphatic sinuses.. Nat Immunol.

[27] Schwab SR, Cyster JG (2007). Finding a way out: lymphocyte egress from lymphoid organs.. Nat Immunol.

[28] Schwab SR, Pereira JP, Matloubian M, Xu Y, Huang Y (2005). Lymphocyte sequestration through S1P lyase inhibition and disruption of S1P gradients.. Science.

[29] Shiow LR, Rosen DB, Brdickova N, Xu Y, An J (2006). CD69 acts downstream of interferon-alpha/beta to inhibit S1P1 and lymphocyte egress from lymphoid organs.. Nature.

[30] Cinamon G, Matloubian M, Lesneski MJ, Xu Y, Low C (2004). Sphingosine 1-phosphate receptor 1 promotes B cell localization in the splenic marginal zone.. Nat Immunol.

[31] Graler MH, Huang MC, Watson S, Goetzl EJ (2005). Immunological effects of transgenic constitutive expression of the type 1 sphingosine 1-phosphate receptor by mouse lymphocytes.. J Immunol.

[32] Kabashima K, Haynes NM, Xu Y, Nutt SL, Allende ML (2006). Plasma cell S1P1 expression determines secondary lymphoid organ retention versus bone marrow tropism.. J Exp Med.

[33] Lo CG, Xu Y, Proia RL, Cyster JG (2005). Cyclical modulation of sphingosine-1-phosphate receptor 1 surface expression during lymphocyte recirculation and relationship to lymphoid organ transit.. J Exp Med.

[34] Matloubian M, Lo CG, Cinamon G, Lesneski MJ, Xu Y (2004). Lymphocyte egress from thymus and peripheral lymphoid organs is dependent on S1P receptor 1.. Nature.

[35] Pham TH, Okada T, Matloubian M, Lo CG, Cyster JG (2008). S1P1 receptor signaling overrides retention mediated by G alpha i-coupled receptors to promote T cell egress.. Immunity.

[36] Gardell SE, Dubin AE, Chun J (2006). Emerging medicinal roles for lysophospholipid signaling.. Trends Mol Med.

[37] Huwiler A, Pfeilschifter J (2008). New players on the center stage: sphingosine 1-phosphate and its receptors as drug targets.. Biochem Pharmacol.

[38] Mandala S, Hajdu R, Bergstrom J, Quackenbush E, Xie J (2002). Alteration of lymphocyte trafficking by sphingosine-1-phosphate receptor agonists.. Science.

[39] Rosen H, Alfonso C, Surh CD, McHeyzer-Williams MG (2003). Rapid induction of medullary thymocyte phenotypic maturation and egress inhibition by nanomolar sphingosine 1-phosphate receptor agonist.. Proc Natl Acad Sci U S A.

[40] Zhang Z, Schluesener HJ (2007). FTY720: a most promising immunosuppressant modulating immune cell functions.. Mini Rev Med Chem.

[41] Zambrowicz BP, Friedrich GA, Buxton EC, Lilleberg SL, Person C (1998). Disruption and sequence identification of 2,000 genes in mouse embryonic stem cells.. Nature.

[42] Yagi H, Kamba R, Chiba K, Soga H, Yaguchi K (2000). Immunosuppressant FTY720 inhibits thymocyte emigration.. Eur J Immunol.

[43] Gabor MJ, Godfrey DI, Scollay R (1997). Recent thymic emigrants are distinct from most medullary thymocytes.. Eur J Immunol.

[44] Brinkmann V, Wilt C, Kristofic C, Nikolova Z, Hof RP (2001). FTY720: dissection of membrane receptor-operated, stereospecific effects on cell migration from receptor-independent antiproliferative and apoptotic effects.. Transplant Proc.

[45] Isoyama N, Takai K, Tsuchida M, Matsumura M, Naito K (2006). Evidence that FTY720 induces rat thymocyte apoptosis.. Transpl Immunol.

[46] Takahama Y (2006). Journey through the thymus: stromal guides for T-cell development and selection.. Nat Rev Immunol.

[47] Pappu R, Schwab SR, Cornelissen I, Pereira JP, Regard JB (2007). Promotion of lymphocyte egress into blood and lymph by distinct sources of sphingosine-1-phosphate.. Science.

[48] Suzuki S, Li XK, Shinomiya T, Enosawa S, Amemiya H (1997). The in vivo induction of lymphocyte apoptosis in MRL-lpr/lpr mice treated with FTY720.. Clin Exp Immunol.

[49] Gon Y, Wood MR, Kiosses WB, Jo E, Sanna MG (2005). S1P3 receptor-induced reorganization of epithelial tight junctions compromises lung barrier integrity and is potentiated by TNF.. Proc Natl Acad Sci U S A.

[50] Lieschke GJ, Stanley E, Grail D, Hodgson G, Sinickas V (1994). Mice lacking both macrophage- and granulocyte-macrophage colony-stimulating factor have macrophages and coexistent osteopetrosis and severe lung disease.. Blood.

[51] Trapnell BC, Whitsett JA, Nakata K (2003). Pulmonary alveolar proteinosis.. N Engl J Med.

[52] Buccoliero R, Palmeri S, Ciarleglio G, Collodoro A, De Santi MM (2007). Increased lung surfactant phosphatidylcholine in patients affected by lysosomal storage diseases.. J Inherit Metab Dis.

[53] Ryu J, Kim HJ, Chang EJ, Huang H, Banno Y (2006). Sphingosine 1-phosphate as a regulator of osteoclast differentiation and osteoclast-osteoblast coupling.. Embo J.

[54] Zambrowicz BP, Abuin A, Ramirez-Solis R, Richter LJ, Piggott J (2003). Wnk1 kinase deficiency lowers blood pressure in mice: a gene-trap screen to identify potential targets for therapeutic intervention.. Proc Natl Acad Sci U S A.

[55] Wattler S, Kelly M, Nehls M (1999). Construction of gene targeting vectors from lambda KOS genomic libraries.. Biotechniques.

[56] O'Gorman S, Dagenais NA, Qian M, Marchuk Y (1997). Protamine-Cre recombinase transgenes efficiently recombine target sequences in the male germ line of mice, but not in embryonic stem cells.. Proc Natl Acad Sci U S A.

[57] Van Veldhoven PP, Mannaerts GP (1991). Subcellular localization and membrane topology of sphingosine-1-phosphate lyase in rat liver.. J Biol Chem.

[58] Yatomi Y, Ruan F, Ohta J, Welch RJ, Hakomori S (1995). Quantitative measurement of sphingosine 1-phosphate in biological samples by acylation with radioactive acetic anhydride.. Anal Biochem.

[59] Murata N, Sato K, Kon J, Tomura H, Okajima F (2000). Quantitative measurement of sphingosine 1-phosphate by radioreceptor-binding assay.. Anal Biochem.

